# Genomes of cressdnaviricots and phixviricots in a freshwater lake sample from Arizona, USA

**DOI:** 10.1128/mra.00920-26

**Published:** 2026-08-28

**Authors:** Joy M. Custer, Simona Kraberger, Grace Levi, Kara Schmidlin, Kristen A. Potter, Dana D. Rosenstein, Elise N. Paietta, Ayla Žuštra, Sydney Millerwise, Arvind Varsani

**Affiliations:** 1 The Biodesign Center for Fundamental and Applied Microbiomics, Center for Evolution and Medicine, School of Life Sciences, Arizona State University 7864, Tempe, Arizona, USA; 2 School of Earth Sciences and Environmental Sustainability, Northern Arizona University 3356, Flagstaff, Arizona, USA; 3 School of Anthropology, University of Arizona 165918, Tucson, Arizona, USA; 4 Structural Biology Research Unit, Department of Integrative Biomedical Sciences, University of Cape Town, Cape Town, South Africa; Queens College Department of Biology, Queens, New York, USA

**Keywords:** *Cressdnaviricota*, *Phixviricota*

## Abstract

A viral metagenomic analysis of a 40 ml water sample from a small freshwater lake near Flagstaff, Arizona, USA, resulted in the identification of 295 circular viral genome sequences. Of these, 126 are members of *Cressdnaviricota* and 169 of *Phixviricota* phyla.

## ANNOUNCEMENT

Single-stranded DNA (ssDNA) virus diversity has been explored in various freshwater lakes and ponds (1–10). As part of an exploratory study to identify viruses within freshwater bodies, a 40ml water sample (in a 50ml tube) from the edge of Lake Mary (35.0806°N, 111.5313°W) near Flagstaff, Arizona, USA, was collected in July 2011.

The sample was processed as described previously (11, 12). In brief, the sample was sequentially filtered through 0.45µm and 0.2µm pore size syringe filters (Sartorius Stedim Biotech, Germany). The filtrate was precipitated using 15% (w/v) PEG 8000 at 4°C overnight. The precipitate was pelleted by centrifugation at 10,000 x g for 10 min and then resuspended in 1 ml of SM buffer (0.1 M NaCl, 50 mM Tris–HCl [pH 7.4]). Two hundred microliters of this filtrate was used for nucleic acid extraction using the High Pure Viral Nucleic Acid Kit (Roche Diagnostics, USA). Circular DNA in the extract was enriched by rolling circle amplification (RCA) using TempliPhi (GE Healthcare, USA). The RCA products were sequenced at Beijing Genomics Institute (Hong Kong, China) on the Illumina HiSeq 2000 platform using their proprietary library preparation workflow with an insert size of 170 bp and 91-bp paired-end reads. The reads were filtered and trimmed with Trimmomatic v0.39 (13) and *de novo* assembled with metaSPAdes v 3.12.0 (14). All bioinformatics tools were used with default settings. Contigs >1000 nts were analyzed using Diamond BLASTx (15) against a RefSeq (16) protein database (release 220). Terminal redundancy was used to identify circular DNA assemblies, and these were annotated using Cenote-taker3 (17–19) and manually checked using Geneious Prime (Dotmatics, USA). We identified 295 circular DNA genomes that fall within the *Cressdnaviricota* (*n* = 126) and *Phixviricota* (*n* = 169) phyla.

The complete genomes of viruses in the phylum *Cressdnaviricota* range in length from 1694 nt to 4672 nt and in GC content from 30.1% to 60.9% (Table 1). Mean coverage depth varied from 4.9× to 2146.59× (Table 1). Of the 126 genomes, only 26 can be classified within currently established families (Table 1): *Adamaviridae* (*n* = 10), *Draupnirviridae* (*n* = 2), *Geplanaviridae* (*n* = 5), *Kanorauviridae* (*n* = 7), *Mahapunaviridae* (*n* = 1), and *Ouroboviridae* (*n* = 1).

**TABLE 1:** Summary of virus genomes identified from the freshwater sample that are members of the *Cressdnaviricota* and *Phixviricota* phyla

Phylum	Class	Order	Family	Accession	% GC	Sequence length (nt)	Mean depth	Read count
*Cressdnaviricota*	–^*a*^	–	–	MW697458	49.40%	1,742	58.36	1,064
*Cressdnaviricota*	–	–	–	MW697459	45.30%	1,748	4.91	91
*Cressdnaviricota*	–	–	–	MW697460	40.70%	1,750	12.36	228
*Cressdnaviricota*	–	–	–	MW697461	51.80%	1,766	1943.56	36,149
*Cressdnaviricota*	–	–	–	MW697462	41.00%	1,777	45.21	851
*Cressdnaviricota*	–	–	–	MW697465	38.80%	1,799	16.54	315
*Cressdnaviricota*	–	–	–	MW697466	47.40%	1,799	10.64	198
*Cressdnaviricota*	–	–	–	MW697467	40.40%	1,803	10.84	209
*Cressdnaviricota*	–	–	–	MW697469	37.90%	1,811	70.94	1,365
*Cressdnaviricota*	–	–	–	MW697471	37.90%	1,895	19.66	400
*Cressdnaviricota*	–	–	–	MW697473	52.50%	1,910	17.57	358
*Cressdnaviricota*	–	–	–	MW697475	50.30%	1,934	17.32	351
*Cressdnaviricota*	–	–	–	MW697476	51.60%	1,948	12.17	249
*Cressdnaviricota*	–	–	–	MW697477	45.40%	1,966	83.29	1,716
*Cressdnaviricota*	–	–	–	MW697480	40.40%	1,987	17.49	369
*Cressdnaviricota*	–	–	–	MW697482	50.90%	2,002	202.84	4,298
*Cressdnaviricota*	–	–	–	MW697483	60.90%	2,041	24.81	532
*Cressdnaviricota*	–	–	–	MW697485	35.50%	2,055	12.99	289
*Cressdnaviricota*	–	–	–	MW697486	52.80%	2,095	425.08	9,535
*Cressdnaviricota*	–	–	–	MW697487	55.60%	2,098	10.55	239
*Cressdnaviricota*	–	–	–	MW697489	53.90%	2,108	14.1	318
*Cressdnaviricota*	–	–	–	MW697490	30.10%	2,128	27.58	623
*Cressdnaviricota*	–	–	–	MW697492	49.00%	2,168	13.18	305
*Cressdnaviricota*	–	–	–	MW697493	46.50%	2,196	680.91	15,960
*Cressdnaviricota*	–	–	–	MW697496	48.40%	2,250	7.88	189
*Cressdnaviricota*	–	–	–	MW697498	42.30%	2,274	52.86	1,286
*Cressdnaviricota*	–	–	–	MW697499	43.10%	2,276	16.08	389
*Cressdnaviricota*	–	–	–	MW697500	48.70%	2,307	61.09	1,507
*Cressdnaviricota*	–	–	–	MW697501	53.60%	2,326	21.46	536
*Cressdnaviricota*	–	–	–	MW697503	52.80%	2,339	37.43	934
*Cressdnaviricota*	–	–	–	MW697504	45.20%	2,349	28.24	710
*Cressdnaviricota*	–	–	–	MW697505	50.90%	2,352	41.64	1,050
*Cressdnaviricota*	–	–	–	MW697506	55.10%	2,361	8.21	209
*Cressdnaviricota*	–	–	–	MW697507	47.00%	2,407	25.57	658
*Cressdnaviricota*	–.	–	–	MW697510	38.80%	2,458	2146.59	56,535
*Cressdnaviricota*	–	–	–	MW697511	47.70%	2,463	54.85	1,442
*Cressdnaviricota*	–	–	–	MW697512	46.20%	2,499	36.85	976
*Cressdnaviricota*	–	–	–	MW697513	41.80%	2,543	77.95	2,120
*Cressdnaviricota*	–	–	–	MW697515	48.40%	2,554	31.7	874
*Cressdnaviricota*	–	–	–	MW697517	50.10%	2,609	80.5	2,249
*Cressdnaviricota*	–	–	–	MW697521	51.80%	2,665	15.16	438
*Cressdnaviricota*	–	–	–	MW697523	52.20%	2,698	85.49	2,493
*Cressdnaviricota*	–	–	–	MW697524	36.00%	2,701	16.45	473
*Cressdnaviricota*	–	–	–	MW697526	41.40%	2,725	128.61	3,756
*Cressdnaviricota*	–	–	–	MW697527	36.70%	2,734	33.14	972
*Cressdnaviricota*	–	–	–	MW697528	51.70%	2,760	170.44	5,054
*Cressdnaviricota*	–	–	–	MW697529	57.50%	2,773	47.98	1,425
*Cressdnaviricota*	–	–	–	MW697531	54.60%	2,813	75.77	2,290
*Cressdnaviricota*	–	–	–	MW697532	50.10%	2,823	192.09	5,846
*Cressdnaviricota*	–	–	–	MW697537	51.10%	2,965	35.64	1,140
*Cressdnaviricota*	–	–	–	MW697538	48.00%	2,967	21.26	683
*Cressdnaviricota*	–	–	–	MW697539	39.60%	2,987	57.19	1,843
*Cressdnaviricota*	–	–	–	MW697540	57.80%	2,992	1715	55,089
*Cressdnaviricota*	–	–	–	MW697541	47.20%	3,005	18.4	594
*Cressdnaviricota*	–	–	–	MW697542	54.40%	3,005	9.95	328
*Cressdnaviricota*	–	–	–	MW697543	41.80%	3,031	81.57	2,667
*Cressdnaviricota*	–	–	–	MW697544	50.20%	3,051	13.23	436
*Cressdnaviricota*	–	–	–	MW697545	50.20%	3,051	14.17	470
*Cressdnaviricota*	–	–	–	MW697546	52.90%	3,058	700.7	23,035
*Cressdnaviricota*	–	–	–	MW697547	52.70%	3,102	69.75	2,314
*Cressdnaviricota*	–	–	–	MW697548	56.80%	3,111	38.79	1,278
*Cressdnaviricota*	–	–	–	MW697549	47.20%	3,115	7.24	241
*Cressdnaviricota*	–	–	–	MW697551	52.90%	3,202	78.34	2,717
*Cressdnaviricota*	–	–	–	MW697552	40.80%	3,216	24.85	863
*Cressdnaviricota*	–	–	–	MW697553	33.20%	3,253	44.84	1,574
*Cressdnaviricota*	–	–	–	MW697554	37.00%	3,272	115.07	4,070
*Cressdnaviricota*	–	–	–	MW697556	55.00%	3,420	874.05	32,545
*Cressdnaviricota*	–	–	–	MW697557	36.00%	3,457	12.78	479
*Cressdnaviricota*	–	–	–	MW697558	38.40%	3,461	39.03	1,460
*Cressdnaviricota*	–	–	–	MW697559	57.20%	3,563	28.88	1,120
*Cressdnaviricota*	–	–	–	MW697560	41.20%	3,589	60.93	2,363
*Cressdnaviricota*	–	–	–	MW697561	42.20%	3,594	20.82	808
*Cressdnaviricota*	–	–	–	MW697562	43.50%	3,687	56.34	2,244
*Cressdnaviricota*	–	–	–	MW697564	44.50%	3,693	10.81	432
*Cressdnaviricota*	–	–	–	MW697566	35.30%	3,730	81.42	3,296
*Cressdnaviricota*	–	–	–	MW697567	42.10%	3,755	9.14	370
*Cressdnaviricota*	–	–	–	MW697568	47.90%	3,835	16.62	692
*Cressdnaviricota*	–	–	–	MW697574	54.40%	3,987	11.56	496
*Cressdnaviricota*	–	–	–	MW697583	48.00%	4,179	157.65	7,178
*Cressdnaviricota*	–	–	–	MW697584	40.70%	4,183	12.06	547
*Cressdnaviricota*	–	–	–	MW697595	38.80%	4,240	31.86	1,460
*Cressdnaviricota*	–	–	–	MW697667	42.50%	4,437	14.28	698
*Cressdnaviricota*	–	–	–	MW697494	41.50%	2,210	7.89	186
*Cressdnaviricota*	*Arfiviricetes*	*Gredzevirales*	*Ouroboviridae*	MW697481	50.40%	1,995	9.97	211
*Cressdnaviricota*	*Arfiviricetes*	*Jormunvirales*	*Draupnirviridae*	MW697472	48.80%	1,900	19.74	398
*Cressdnaviricota*	*Arfiviricetes*	*Jormunvirales*	*Draupniviridae*	MW697478	43.20%	1,969	26.22	547
*Cressdnaviricota*	*Arfiviricetes*	*Mulpavirales*	–	MW697457	44.50%	1,694	15.34	271
*Cressdnaviricota*	*Arfiviricetes*	*Mulpavirales*	–	MW697468	43.20%	1,809	21.92	418
*Cressdnaviricota*	*Arfiviricetes*	*Mulpavirales*	–	MW697470	45.70%	1,893	28.01	557
*Cressdnaviricota*	*Arfiviricetes*	*Mulpavirales*	–	MW697479	44.90%	1,983	114.85	2,408
*Cressdnaviricota*	*Arfiviricetes*	*Mulpavirales*	–	MW697484	47.00%	2,051	1027.14	22,226
*Cressdnaviricota*	*Arfiviricetes*	*Mulpavirales*	–	MW697491	47.10%	2,134	8.57	193
*Cressdnaviricota*	*Arfiviricetes*	*Mulpavirales*	–	MW697495	34.10%	2,227	16.29	385
*Cressdnaviricota*	*Arfiviricetes*	*Mulpavirales*	–	MW697563	46.70%	3,691	32.56	1,298
*Cressdnaviricota*	*Arfiviricetes*	*Mulpavirales*	–	MW697657	40.40%	4,424	132.1	6,379
*Cressdnaviricota*	*Arfiviricetes*	*Rohanvirales*	–	MW697463	39.00%	1,788	38.89	741
*Cressdnaviricota*	*Arfiviricetes*	*Rohanvirales*	–	MW697464	39.10%	1,799	12.14	233
*Cressdnaviricota*	*Arfiviricetes*	*Rohanvirales*	–	MW697474	36.20%	1,913	59.56	1,206
*Cressdnaviricota*	*Arfiviricetes*	*Rohanvirales*	–	MW697569	45.90%	3,868	309.86	13,014
*Cressdnaviricota*	*Arfiviricetes*	*Rohanvirales*	–	MW697570	48.00%	3,889	9.14	383
*Cressdnaviricota*	*Arfiviricetes*	*Rohanvirales*	–	MW697579	46.70%	4,140	151.75	6,851
*Cressdnaviricota*	*Arfiviricetes*	*Rohanvirales*	*Adamaviridae*	MW697508	42.40%	2,413	21.13	546
*Cressdnaviricota*	*Arfiviricetes*	*Rohanvirales*	*Adamaviridae*	MW697520	38.30%	2,663	354.23	10,099
*Cressdnaviricota*	*Arfiviricetes*	*Rohanvirales*	*Adamaviridae*	MW697522	44.90%	2,666	15.34	438
*Cressdnaviricota*	*Arfiviricetes*	*Rohanvirales*	*Adamaviridae*	MW697534	47.90%	2,842	22.14	675
*Cressdnaviricota*	*Arfiviricetes*	*Rohanvirales*	*Adamaviridae*	MW697565	47.70%	3,711	27.56	1,087
*Cressdnaviricota*	*Arfiviricetes*	*Rohanvirales*	*Adamaviridae*	MW697596	40.00%	4,243	72.55	3,340
*Cressdnaviricota*	*Arfiviricetes*	*Rohanvirales*	*Adamaviridae*	MW697623	44.80%	4,328	27.58	1,304
*Cressdnaviricota*	*Arfiviricetes*	*Rohanvirales*	*Adamaviridae*	MW697687	43.20%	4,496	35.09	1,719
*Cressdnaviricota*	*Arfiviricetes*	*Rohanvirales*	*Adamaviridae*	MW697694	40.50%	4,512	17.83	876
*Cressdnaviricota*	*Arfiviricetes*	*Rohanvirales*	*Adamaviridae*	MW697732	37.90%	4,672	26	1,244
*Cressdnaviricota*	*Arfiviricetes*	*Saturnivirales*	*Kanorauviridae*	MW697497	43.30%	2,268	12.15	296
*Cressdnaviricota*	*Arfiviricetes*	*Saturnivirales*	*Kanorauviridae*	MW697509	42.70%	2,448	7.57	197
*Cressdnaviricota*	*Arfiviricetes*	*Saturnivirales*	*Kanorauviridae*	MW697516	41.50%	2,579	11.93	328
*Cressdnaviricota*	*Arfiviricetes*	*Saturnivirales*	*Kanorauviridae*	MW697518	45.50%	2,619	250.62	7,041
*Cressdnaviricota*	*Arfiviricetes*	*Saturnivirales*	*Kanorauviridae*	MW697525	39.20%	2,716	34.06	989
*Cressdnaviricota*	*Arfiviricetes*	*Saturnivirales*	*Kanorauviridae*	MW697550	40.10%	3,154	20.39	690
*Cressdnaviricota*	*Arfiviricetes*	*Saturnivirales*	*Kanorauviridae*	MW697555	41.40%	3,277	48.1	1,702
*Cressdnaviricota*	*Arfiviricetes*	*Saturnivirales*	*Mahapunaviridae*	MW697488	46.00%	2,106	11.3	253
*Cressdnaviricota*	*Repensiviricetes*	*Geplafuvirales*	–	MW697519	41.70%	2,663	12.54	357
*Cressdnaviricota*	*Repensiviricetes*	*Geplafuvirales*	–	MW697533	46.60%	2,839	52.87	1,616
*Cressdnaviricota*	*Repensiviricetes*	*Geplafuvirales*	*Geplanaviridae*	MW697502	51.50%	2,332	5.76	143
*Cressdnaviricota*	*Repensiviricetes*	*Geplafuvirales*	*Geplanaviridae*	MW697514	41.10%	2,553	368.78	10,092
*Cressdnaviricota*	*Repensiviricetes*	*Geplafuvirales*	*Geplanaviridae*	MW697530	33.80%	2,799	66.99	2,020
*Cressdnaviricota*	*Repensiviricetes*	*Geplafuvirales*	*Geplanaviridae*	MW697535	40.40%	2,899	94.64	2,950
*Cressdnaviricota*	*Repensiviricetes*	*Geplafuvirales*	*Geplanaviridae*	MW697536	40.30%	2,942	36.35	1,154
*Phixviricota*	*Microviricetes*	*Amoyvirales*	*Mazaviridae*	MW697573	64.40%	3,976	13.33	580
*Phixviricota*	*Microviricetes*	*Amoyvirales*	*Mazaviridae*	MW697746	60.20%	4,916	50.28	2,699
*Phixviricota*	*Microviricetes*	*Bullavirales*	*Pequenoviridae*	MW697748	54.90%	5,938	56.56	3,681
*Phixviricota*	*Microviricetes*	*Gokushovirales*	*Betagokushoviridae*	MW697578	56.50%	4,136	71.23	3,185
*Phixviricota*	*Microviricetes*	*Gokushovirales*	*Betagokushoviridae*	MW697585	41.10%	4,201	150.92	6,911
*Phixviricota*	*Microviricetes*	*Gokushovirales*	*Betagokushoviridae*	MW697586	47.60%	4,203	87.32	3,024
*Phixviricota*	*Microviricetes*	*Gokushovirales*	*Betagokushoviridae*	MW697590	42.30%	4,228	127.57	5,817
*Phixviricota*	*Microviricetes*	*Gokushovirales*	*Betagokushoviridae*	MW697592	43.10%	4,232	241.98	11,161
*Phixviricota*	*Microviricetes*	*Gokushovirales*	*Betagokushoviridae*	MW697594	42.60%	4,238	395.37	18,244
*Phixviricota*	*Microviricetes*	*Gokushovirales*	*Betagokushoviridae*	MW697598	45.50%	4,268	125.91	5,838
*Phixviricota*	*Microviricetes*	*Gokushovirales*	*Betagokushoviridae*	MW697601	55.20%	4,275	31.55	1,462
*Phixviricota*	*Microviricetes*	*Gokushovirales*	*Betagokushoviridae*	MW697602	41.90%	4,278	125.74	5,863
*Phixviricota*	*Microviricetes*	*Gokushovirales*	*Betagokushoviridae*	MW697603	41.70%	4,279	852.71	39,761
*Phixviricota*	*Microviricetes*	*Gokushovirales*	*Betagokushoviridae*	MW697605	42.10%	4,288	793.76	37,059
*Phixviricota*	*Microviricetes*	*Gokushovirales*	*Betagokushoviridae*	MW697607	46.50%	4,291	47.62	2,229
*Phixviricota*	*Microviricetes*	*Gokushovirales*	*Betagokushoviridae*	MW697609	44.00%	4,298	189.05	8,885
*Phixviricota*	*Microviricetes*	*Gokushovirales*	*Betagokushoviridae*	MW697611	48.50%	4,302	151.82	7,107
*Phixviricota*	*Microviricetes*	*Gokushovirales*	*Betagokushoviridae*	MW697615	43.10%	4,314	70.7	3,316
*Phixviricota*	*Microviricetes*	*Gokushovirales*	*Betagokushoviridae*	MW697616	41.50%	4,320	22.18	1,042
*Phixviricota*	*Microviricetes*	*Gokushovirales*	*Betagokushoviridae*	MW697619	38.90%	4,325	8.91	419
*Phixviricota*	*Microviricetes*	*Gokushovirales*	*Betagokushoviridae*	MW697620	41.50%	4,327	7.81	369
*Phixviricota*	*Microviricetes*	*Gokushovirales*	*Betagokushoviridae*	MW697622	57.30%	4,328	60.75	2,878
*Phixviricota*	*Microviricetes*	*Gokushovirales*	*Betagokushoviridae*	MW697625	47.70%	4,341	239.3	11,307
*Phixviricota*	*Microviricetes*	*Gokushovirales*	*Betagokushoviridae*	MW697626	55.00%	4,344	28.23	1,341
*Phixviricota*	*Microviricetes*	*Gokushovirales*	*Betagokushoviridae*	MW697627	55.10%	4,345	73.93	3,501
*Phixviricota*	*Microviricetes*	*Gokushovirales*	*Betagokushoviridae*	MW697628	47.40%	4,347	10.13	479
*Phixviricota*	*Microviricetes*	*Gokushovirales*	*Betagokushoviridae*	MW697629	57.10%	4,349	21.06	999
*Phixviricota*	*Microviricetes*	*Gokushovirales*	*Betagokushoviridae*	MW697630	40.80%	4,350	61.65	2,919
*Phixviricota*	*Microviricetes*	*Gokushovirales*	*Betagokushoviridae*	MW697632	54.70%	4,356	73.2	3,476
*Phixviricota*	*Microviricetes*	*Gokushovirales*	*Betagokushoviridae*	MW697633	48.20%	4,369	665.06	31,630
*Phixviricota*	*Microviricetes*	*Gokushovirales*	*Betagokushoviridae*	MW697634	42.60%	4,373	83.29	3,972
*Phixviricota*	*Microviricetes*	*Gokushovirales*	*Betagokushoviridae*	MW697635	43.90%	4,373	33.02	1,573
*Phixviricota*	*Microviricetes*	*Gokushovirales*	*Betagokushoviridae*	MW697636	47.50%	4,375	18.18	863
*Phixviricota*	*Microviricetes*	*Gokushovirales*	*Betagokushoviridae*	MW697638	41.70%	4,379	20.31	971
*Phixviricota*	*Microviricetes*	*Gokushovirales*	*Betagokushoviridae*	MW697639	42.00%	4,387	55.72	2,665
*Phixviricota*	*Microviricetes*	*Gokushovirales*	*Betagokushoviridae*	MW697640	41.60%	4,391	17.78	850
*Phixviricota*	*Microviricetes*	*Gokushovirales*	*Betagokushoviridae*	MW697641	46.70%	4,393	11.84	566
*Phixviricota*	*Microviricetes*	*Gokushovirales*	*Betagokushoviridae*	MW697643	47.00%	4,398	174	8,345
*Phixviricota*	*Microviricetes*	*Gokushovirales*	*Betagokushoviridae*	MW697644	41.30%	4,400	36.07	1,730
*Phixviricota*	*Microviricetes*	*Gokushovirales*	*Betagokushoviridae*	MW697645	45.50%	4,403	308.02	14,798
*Phixviricota*	*Microviricetes*	*Gokushovirales*	*Betagokushoviridae*	MW697646	46.50%	4,406	127.99	6,138
*Phixviricota*	*Microviricetes*	*Gokushovirales*	*Betagokushoviridae*	MW697648	46.80%	4,408	126.34	6,064
*Phixviricota*	*Microviricetes*	*Gokushovirales*	*Betagokushoviridae*	MW697649	54.20%	4,409	11.72	563
*Phixviricota*	*Microviricetes*	*Gokushovirales*	*Betagokushoviridae*	MW697650	43.10%	4,411	53.07	2,546
*Phixviricota*	*Microviricetes*	*Gokushovirales*	*Betagokushoviridae*	MW697652	54.60%	4,412	17.72	851
*Phixviricota*	*Microviricetes*	*Gokushovirales*	*Betagokushoviridae*	MW697653	45.70%	4,413	27.86	1,345
*Phixviricota*	*Microviricetes*	*Gokushovirales*	*Betagokushoviridae*	MW697655	42.30%	4,420	45.7	2,195
*Phixviricota*	*Microviricetes*	*Gokushovirales*	*Betagokushoviridae*	MW697656	55.10%	4,421	92.29	4,462
*Phixviricota*	*Microviricetes*	*Gokushovirales*	*Betagokushoviridae*	MW697658	40.00%	4,424	98.47	4,747
*Phixviricota*	*Microviricetes*	*Gokushovirales*	*Betagokushoviridae*	MW697659	47.90%	4,425	20.8	1,011
*Phixviricota*	*Microviricetes*	*Gokushovirales*	*Betagokushoviridae*	MW697660	48.90%	4,425	12.58	610
*Phixviricota*	*Microviricetes*	*Gokushovirales*	*Betagokushoviridae*	MW697661	59.30%	4,428	35.77	1,722
*Phixviricota*	*Microviricetes*	*Gokushovirales*	*Betagokushoviridae*	MW697662	54.00%	4,432	96.17	4,637
*Phixviricota*	*Microviricetes*	*Gokushovirales*	*Betagokushoviridae*	MW697663	54.40%	4,433	169.78	8,187
*Phixviricota*	*Microviricetes*	*Gokushovirales*	*Betagokushoviridae*	MW697664	44.70%	4,433	51.67	2,498
*Phixviricota*	*Microviricetes*	*Gokushovirales*	*Betagokushoviridae*	MW697665	43.40%	4,437	204.55	9,890
*Phixviricota*	*Microviricetes*	*Gokushovirales*	*Betagokushoviridae*	MW697666	54.70%	4,437	18.52	895
*Phixviricota*	*Microviricetes*	*Gokushovirales*	*Betagokushoviridae*	MW697669	42.00%	4,442	50.27	2,449
*Phixviricota*	*Microviricetes*	*Gokushovirales*	*Betagokushoviridae*	MW697670	55.10%	4,445	261.03	12,669
*Phixviricota*	*Microviricetes*	*Gokushovirales*	*Betagokushoviridae*	MW697671	47.30%	4,453	36.31	1,754
*Phixviricota*	*Microviricetes*	*Gokushovirales*	*Betagokushoviridae*	MW697672	41.70%	4,455	22.75	1,108
*Phixviricota*	*Microviricetes*	*Gokushovirales*	*Betagokushoviridae*	MW697673	45.10%	4,458	9.28	452
*Phixviricota*	*Microviricetes*	*Gokushovirales*	*Betagokushoviridae*	MW697675	42.40%	4,463	57.13	2,781
*Phixviricota*	*Microviricetes*	*Gokushovirales*	*Betagokushoviridae*	MW697676	42.00%	4,465	1632.08	79,387
*Phixviricota*	*Microviricetes*	*Gokushovirales*	*Betagokushoviridae*	MW697677	44.30%	4,465	26.29	1,278
*Phixviricota*	*Microviricetes*	*Gokushovirales*	*Betagokushoviridae*	MW697678	50.20%	4,466	39.23	1,902
*Phixviricota*	*Microviricetes*	*Gokushovirales*	*Betagokushoviridae*	MW697679	46.40%	4,467	668.3	32,562
*Phixviricota*	*Microviricetes*	*Gokushovirales*	*Betagokushoviridae*	MW697680	41.80%	4,469	105.92	5,173
*Phixviricota*	*Microviricetes*	*Gokushovirales*	*Betagokushoviridae*	MW697681	48.10%	4,470	133.46	6,477
*Phixviricota*	*Microviricetes*	*Gokushovirales*	*Betagokushoviridae*	MW697682	46.00%	4,471	61	2,981
*Phixviricota*	*Microviricetes*	*Gokushovirales*	*Betagokushoviridae*	MW697683	54.40%	4,476	69	3,377
*Phixviricota*	*Microviricetes*	*Gokushovirales*	*Betagokushoviridae*	MW697684	48.30%	4,477	154.88	7,564
*Phixviricota*	*Microviricetes*	*Gokushovirales*	*Betagokushoviridae*	MW697685	46.90%	4,482	705.67	34,591
*Phixviricota*	*Microviricetes*	*Gokushovirales*	*Betagokushoviridae*	MW697686	41.40%	4,490	13.93	680
*Phixviricota*	*Microviricetes*	*Gokushovirales*	*Betagokushoviridae*	MW697688	44.40%	4,497	39.65	1,945
*Phixviricota*	*Microviricetes*	*Gokushovirales*	*Betagokushoviridae*	MW697690	49.60%	4,500	17.24	844
*Phixviricota*	*Microviricetes*	*Gokushovirales*	*Betagokushoviridae*	MW697691	48.30%	4,502	9.27	455
*Phixviricota*	*Microviricetes*	*Gokushovirales*	*Betagokushoviridae*	MW697692	59.00%	4,509	131.66	6,454
*Phixviricota*	*Microviricetes*	*Gokushovirales*	*Betagokushoviridae*	MW697693	39.60%	4,510	16.7	816
*Phixviricota*	*Microviricetes*	*Gokushovirales*	*Betagokushoviridae*	MW697695	43.80%	4,517	13.65	673
*Phixviricota*	*Microviricetes*	*Gokushovirales*	*Betagokushoviridae*	MW697696	50.90%	4,519	88.36	4,350
*Phixviricota*	*Microviricetes*	*Gokushovirales*	*Betagokushoviridae*	MW697697	43.80%	4,520	17.53	866
*Phixviricota*	*Microviricetes*	*Gokushovirales*	*Betagokushoviridae*	MW697698	45.30%	4,521	20.6	1,016
*Phixviricota*	*Microviricetes*	*Gokushovirales*	*Betagokushoviridae*	MW697699	60.30%	4,530	29.85	1471
*Phixviricota*	*Microviricetes*	*Gokushovirales*	*Betagokushoviridae*	MW697700	50.90%	4,534	21.07	1035
*Phixviricota*	*Microviricetes*	*Gokushovirales*	*Betagokushoviridae*	MW697702	59.90%	4,542	22.02	1088
*Phixviricota*	*Microviricetes*	*Gokushovirales*	*Betagokushoviridae*	MW697703	60.00%	4,542	24.37	1209
*Phixviricota*	*Microviricetes*	*Gokushovirales*	*Betagokushoviridae*	MW697704	47.30%	4,542	12.87	638
*Phixviricota*	*Microviricetes*	*Gokushovirales*	*Betagokushoviridae*	MW697705	44.00%	4,543	87.49	4340
*Phixviricota*	*Microviricetes*	*Gokushovirales*	*Betagokushoviridae*	MW697706	39.00%	4,546	11.9	592
*Phixviricota*	*Microviricetes*	*Gokushovirales*	*Betagokushoviridae*	MW697707	45.00%	4,549	74.97	3723
*Phixviricota*	*Microviricetes*	*Gokushovirales*	*Betagokushoviridae*	MW697708	43.10%	4,553	11.59	578
*Phixviricota*	*Microviricetes*	*Gokushovirales*	*Betagokushoviridae*	MW697709	48.20%	4,555	128.02	6387
*Phixviricota*	*Microviricetes*	*Gokushovirales*	*Betagokushoviridae*	MW697710	40.80%	4,555	107.56	5348
*Phixviricota*	*Microviricetes*	*Gokushovirales*	*Betagokushoviridae*	MW697711	44.90%	4,577	97.31	4818
*Phixviricota*	*Microviricetes*	*Gokushovirales*	*Betagokushoviridae*	MW697712	48.70%	4,581	9.71	485
*Phixviricota*	*Microviricetes*	*Gokushovirales*	*Betagokushoviridae*	MW697713	45.90%	4,584	33.64	1687
*Phixviricota*	*Microviricetes*	*Gokushovirales*	*Betagokushoviridae*	MW697714	52.10%	4,587	15.06	754
*Phixviricota*	*Microviricetes*	*Gokushovirales*	*Betagokushoviridae*	MW697715	40.00%	4,587	13.6	678
*Phixviricota*	*Microviricetes*	*Gokushovirales*	*Betagokushoviridae*	MW697716	43.90%	4,595	34.71	1741
*Phixviricota*	*Microviricetes*	*Gokushovirales*	*Betagokushoviridae*	MW697717	44.00%	4,605	338.33	17,019
*Phixviricota*	*Microviricetes*	*Gokushovirales*	*Betagokushoviridae*	MW697718	41.40%	4,605	22.75	1141
*Phixviricota*	*Microviricetes*	*Gokushovirales*	*Betagokushoviridae*	MW697720	44.20%	4,611	57.11	2869
*Phixviricota*	*Microviricetes*	*Gokushovirales*	*Betagokushoviridae*	MW697721	45.50%	4,612	116.2	5840
*Phixviricota*	*Microviricetes*	*Gokushovirales*	*Betagokushoviridae*	MW697722	56.90%	4,626	166.41	8409
*Phixviricota*	*Microviricetes*	*Gokushovirales*	*Betagokushoviridae*	MW697723	59.90%	4,635	14.12	714
*Phixviricota*	*Microviricetes*	*Gokushovirales*	*Betagokushoviridae*	MW697724	40.00%	4,638	26.41	1336
*Phixviricota*	*Microviricetes*	*Gokushovirales*	*Betagokushoviridae*	MW697726	46.80%	4,644	14.92	753
*Phixviricota*	*Microviricetes*	*Gokushovirales*	*Betagokushoviridae*	MW697727	41.10%	4,645	118.89	6007
*Phixviricota*	*Microviricetes*	*Gokushovirales*	*Betagokushoviridae*	MW697728	57.50%	4,658	12.22	623
*Phixviricota*	*Microviricetes*	*Gokushovirales*	*Betagokushoviridae*	MW697729	43.30%	4,659	1043.49	52,984
*Phixviricota*	*Microviricetes*	*Gokushovirales*	*Betagokushoviridae*	MW697730	39.60%	4,659	10.11	510
*Phixviricota*	*Microviricetes*	*Gokushovirales*	*Betagokushoviridae*	MW697731	53.40%	4,667	10.85	551
*Phixviricota*	*Microviricetes*	*Gokushovirales*	*Betagokushoviridae*	MW697734	50.60%	4,684	637.02	32,593
*Phixviricota*	*Microviricetes*	*Gokushovirales*	*Betagokushoviridae*	MW697735	45.60%	4,687	21.41	1089
*Phixviricota*	*Microviricetes*	*Gokushovirales*	*Betagokushoviridae*	MW697736	46.60%	4,694	191.1	9806
*Phixviricota*	*Microviricetes*	*Gokushovirales*	*Betagokushoviridae*	MW697737	49.80%	4,735	29.88	1547
*Phixviricota*	*Microviricetes*	*Gokushovirales*	*Betagokushoviridae*	MW697738	58.00%	4,735	33.54	1731
*Phixviricota*	*Microviricetes*	*Gokushovirales*	*Betagokushoviridae*	MW697739	44.40%	4,739	1229.63	63,629
*Phixviricota*	*Microviricetes*	*Gokushovirales*	*Betagokushoviridae*	MW697740	39.10%	4,739	24.76	1284
*Phixviricota*	*Microviricetes*	*Gokushovirales*	*Betagokushoviridae*	MW697741	60.70%	4,750	112.41	5808
*Phixviricota*	*Microviricetes*	*Gokushovirales*	*Betagokushoviridae*	MW697742	57.80%	4,804	40.78	2132
*Phixviricota*	*Microviricetes*	*Gokushovirales*	*Betagokushoviridae*	MW697743	58.00%	4,808	13.17	688
*Phixviricota*	*Microviricetes*	*Gokushovirales*	*Betagokushoviridae*	MW697744	48.80%	4,830	74.72	3948
*Phixviricota*	*Microviricetes*	*Gokushovirales*	*Betagokushoviridae*	MW697745	57.80%	4,836	94.81	5000
*Phixviricota*	*Microviricetes*	*Gokushovirales*	*Betagokushoviridae*	MW697747	40.30%	5,176	282.63	16,012
*Phixviricota*	*Microviricetes*	*Gokushovirales*	*Groupodiviridae*	MW697576	40.60%	4,103	29.74	1331
*Phixviricota*	*Microviricetes*	*Gokushovirales*	*Groupodiviridae*	MW697580	55.80%	4,150	15.87	717
*Phixviricota*	*Microviricetes*	*Gokushovirales*	*Groupodiviridae*	MW697587	51.20%	4,214	26.21	1204
*Phixviricota*	*Microviricetes*	*Gokushovirales*	*Groupodiviridae*	MW697599	54.50%	4,270	767.09	35,651
*Phixviricota*	*Microviricetes*	*Gokushovirales*	*Groupodiviridae*	MW697600	62.20%	4,271	484.79	22,553
*Phixviricota*	*Microviricetes*	*Gokushovirales*	*Groupodiviridae*	MW697604	55.70%	4,287	16.7	784
*Phixviricota*	*Microviricetes*	*Gokushovirales*	*Groupodiviridae*	MW697606	52.90%	4,290	108.08	5074
*Phixviricota*	*Microviricetes*	*Gokushovirales*	*Groupodiviridae*	MW697617	44.40%	4,322	283.88	13,382
*Phixviricota*	*Microviricetes*	*Gokushovirales*	*Groupodiviridae*	MW697618	45.40%	4,325	51.03	2412
*Phixviricota*	*Microviricetes*	*Gokushovirales*	*Groupodiviridae*	MW697621	56.70%	4,328	280.02	13,219
*Phixviricota*	*Microviricetes*	*Gokushovirales*	*Groupodiviridae*	MW697631	40.90%	4,355	241.67	11,425
*Phixviricota*	*Microviricetes*	*Gokushovirales*	*Groupodiviridae*	MW697637	52.70%	4,378	8.9	423
*Phixviricota*	*Microviricetes*	*Gokushovirales*	*Groupodiviridae*	MW697642	53.60%	4,397	15.16	732
*Phixviricota*	*Microviricetes*	*Gokushovirales*	*Groupodiviridae*	MW697647	55.30%	4,408	206.22	9910
*Phixviricota*	*Microviricetes*	*Gokushovirales*	*Groupodiviridae*	MW697654	51.00%	4,414	35.49	1719
*Phixviricota*	*Microviricetes*	*Gokushovirales*	*Groupodiviridae*	MW697668	53.00%	4,441	225.27	10,975
*Phixviricota*	*Microviricetes*	*Gokushovirales*	*Groupodiviridae*	MW697689	49.40%	4,497	20.26	992
*Phixviricota*	*Microviricetes*	*Gokushovirales*	*Groupodiviridae*	MW697701	55.90%	4,538	17.72	876
*Phixviricota*	*Microviricetes*	*Gokushovirales*	*Pichoviridae*	MW697572	36.50%	3,963	40.82	1754
*Phixviricota*	*Microviricetes*	*Gokushovirales*	*Pichoviridae*	MW697577	37.60%	4134	73.11	3303
*Phixviricota*	*Microviricetes*	*Gokushovirales*	*Pichoviridae*	MW697582	52.00%	4163	185.11	8393
*Phixviricota*	*Microviricetes*	*Gokushovirales*	*Pichoviridae*	MW697597	38.50%	4251	14.12	654
*Phixviricota*	*Microviricetes*	*Gokushovirales*	*Pichoviridae*	MW697608	43.50%	4296	20.31	951
*Phixviricota*	*Microviricetes*	*Gokushovirales*	*Pichoviridae*	MW697610	41.30%	4298	17.57	825
*Phixviricota*	*Microviricetes*	*Gokushovirales*	*Pichoviridae*	MW697614	37.90%	4311	13.65	639
*Phixviricota*	*Microviricetes*	*Gokushovirales*	*Pichoviridae*	MW697624	43.60%	4336	294.54	13,912
*Phixviricota*	*Microviricetes*	*Gokushovirales*	*Pichoviridae*	MW697674	38.40%	4460	51.34	2493
*Phixviricota*	*Microviricetes*	*Gokushovirales*	*Pichoviridae*	MW697719	44.30%	4607	28.63	1441
*Phixviricota*	*Microviricetes*	*Gokushovirales*	*Pichoviridae*	MW697725	36.90%	4640	32.59	1647
*Phixviricota*	*Microviricetes*	*Gokushovirales*	*Sukshmaviridae*	MW697571	46.40%	3923	495.78	21,154
*Phixviricota*	*Microviricetes*	*Gokushovirales*	*Sukshmaviridae*	MW697575	35.10%	4007	8.49	368
*Phixviricota*	*Microviricetes*	*Gokushovirales*	*Sukshmaviridae*	MW697581	35.80%	4161	99.64	4512
*Phixviricota*	*Microviricetes*	*Gokushovirales*	*Sukshmaviridae*	MW697588	37.30%	4221	16.21	746
*Phixviricota*	*Microviricetes*	*Gokushovirales*	*Sukshmaviridae*	MW697589	48.30%	4226	44.17	2039
*Phixviricota*	*Microviricetes*	*Gokushovirales*	*Sukshmaviridae*	MW697591	38.50%	4232	13.49	623
*Phixviricota*	*Microviricetes*	*Gokushovirales*	*Sukshmaviridae*	MW697593	39.10%	4235	159.99	7373
*Phixviricota*	*Microviricetes*	*Gokushovirales*	*Sukshmaviridae*	MW697612	36.40%	4308	24.22	1137
*Phixviricota*	*Microviricetes*	*Gokushovirales*	*Sukshmaviridae*	MW697613	48.60%	4310	70.19	3292
*Phixviricota*	*Microviricetes*	*Gokushovirales*	*Sukshmaviridae*	MW697651	52.00%	4411	24.65	1182
*Phixviricota*	*Microviricetes*	*Gokushovirales*	*Sukshmaviridae*	MW697733	53.80%	4679	74.88	3826
*Phixviricota*	*Microviricetes*	*Secretvirales*	*Chicoviridae*	MW697749	58.90%	6048	38.06	2521
*Phixviricota*	*Microviricetes*	*Secretvirales*	*Chicoviridae*	MW697750	52.00%	6243	81.6	5588
*Phixviricota*	*Microviricetes*	*Secretvirales*	*Chicoviridae*	MW697751	61.10%	6258	11.21	771

^*a*^ –, unclassified.

The 169 complete genomes of viruses in the phylum *Phixviricota* range in length from 3923nt to 6258nt and in GC content from 35.1% to 64.4% (Table 1). Mean coverage depth varied from 7.8× to 1632.1× (Table 1). All these virus genomes fall within established families (Table 1): *Betagokushoviridae* (*n* = 123), *Chicoviridae* (*n* = 3), *Groupodiviridae* (*n* = 18), *Mazaviridae* (*n* = 2), *Pequenoviridae* (*n* = 1), *Pichoviridae* (*n* = 11) and *Sukshmaviridae* (*n* = 11).

The identification of 295 genomes of ssDNA viruses within a 50ml freshwater sample highlights the significant viral diversity that exists within freshwater bodies. Furthermore, of all these virus genomes only one (a cressdnaviricot) can be assigned to an established species (Arizlama virus AZLM_985, MW697472; family *Draupnirviridae*; species *Viratusivirus arilasis*). This is not surprising given that these freshwater bodies support diverse animals (including fish, amphibians, invertebrates), plants, and various microorganisms (including bacteria and protists). Long-term viral monitoring of freshwater bodies may shed light on the temporal dynamics of these viral communities.

## Data Availability

These sequences are available for *Cressdnaviricota* under accessions MW697457–MW697732 and *Microviricetes* under accessions MW697571–MW697751 in GenBank. The raw reads are deposited in BioProject number PRJNA702105 with SRA number SRR13721232.

## References

[1] Aguirre de Cárcer D, López-Bueno A, Pearce DA, Alcamí A. 2015. Biodiversity and distribution of polar freshwater DNA viruses. Sci Adv 1:e1400127. doi:10.1126/sciadv.1400127 PMC4640604 26601189

[2] Dayaram A, Galatowitsch ML, Argüello-Astorga GR, van Bysterveldt K, Kraberger S, Stainton D, Harding JS, Roumagnac P, Martin DP, Lefeuvre P, Varsani A. 2016. Diverse circular replication-associated protein encoding viruses circulating in invertebrates within a lake ecosystem. Infect Genet Evol 39:304–316. doi:10.1016/j.meegid.2016.02.011 26873065

[3] Gezon NR, Haywick DW, Sanders JM, Hewson I, Strychar KB. 2020. Circular Rep encoding single stranded (CRESS) DNA virus-like sequences detected in quagga mussels (*Dreissena rostriformis bugensis*) and sediments from the central Lake Michigan benthos. J Great Lakes Res 46:302–310. doi:10.1016/j.jglr.2020.01.011

[4] Laanto E, Mäntynen S, De Colibus L, Marjakangas J, Gillum A, Stuart DI, Ravantti JJ, Huiskonen JT, Sundberg L-R. 2017. Virus found in a boreal lake links ssDNA and dsDNA viruses. Proc Natl Acad Sci USA 114:8378–8383. doi:10.1073/pnas.1703834114 PMC5547622 28716906

[5] Lund MC, Hopkins A, Dayaram A, Galatowitsch ML, Stainton D, Harding JS, Lefeuvre P, Zhu Q, Kraberger S, Varsani A. 2024. Diverse microviruses circulating in invertebrates within a lake ecosystem. J Gen Virol 105:11. doi:10.1099/jgv.0.002049 39565345

[6] Malki K, Rosario K, Sawaya NA, Székely AJ, Tisza MJ, Breitbart M. 2020. Prokaryotic and viral community composition of freshwater springs In Florida, USA. mBio 11:e00436-20. doi:10.1128/mBio.00436-20 PMC7157768 32265327

[7] Zawar-Reza P, Argüello-Astorga GR, Kraberger S, Julian L, Stainton D, Broady PA, Varsani A. 2014. Diverse small circular single-stranded DNA viruses identified in a freshwater pond on the McMurdo Ice Shelf (Antarctica). Infect Genet Evol 26:132–138. doi:10.1016/j.meegid.2014.05.018 24859088

[8] Zhong X, Guidoni B, Jacas L, Jacquet S. 2015. Structure and diversity of ssDNA Microviridae viruses in two peri-alpine lakes (Annecy and Bourget, France). Res Microbiol 166:644–654. doi:10.1016/j.resmic.2015.07.003 26226335

[9] Zhou Z, Tran PQ, Martin C, Rohwer RR, Baker BJ, McMahon KD, Anantharaman K. 2025. Unravelling viral ecology and evolution over 20 years in a freshwater lake. Nat Microbiol 10:231–245. doi:10.1038/s41564-024-01876-7 39753667

[10] Roux S, Enault F, Robin A, Ravet V, Personnic S, Theil S, Colombet J, Sime-Ngando T, Debroas D. 2012. Assessing the diversity and specificity of two freshwater viral communities through metagenomics. PLoS One 7:e33641. doi:10.1371/journal.pone.0033641 PMC3303852 22432038

[11] Kraberger S, Argüello-Astorga GR, Greenfield LG, Galilee C, Law D, Martin DP, Varsani A. 2015. Characterisation of a diverse range of circular replication-associated protein encoding DNA viruses recovered from a sewage treatment oxidation pond. Infect Genet Evol 31:73–86. doi:10.1016/j.meegid.2015.01.001 25583447

[12] Kraberger S, Schreck J, Galilee C, Varsani A. 2021. Genome sequences of microviruses identified in a sample from a sewage treatment oxidation pond. Microbiol Resour Announc 10:19. doi:10.1128/MRA.00373-21 PMC8142586 33986100

[13] Bolger AM, Lohse M, Usadel B. 2014. Trimmomatic: a flexible trimmer for Illumina sequence data. Bioinformatics 30:2114–2120. doi:10.1093/bioinformatics/btu170 PMC4103590 24695404

[14] Bankevich A, Nurk S, Antipov D, Gurevich AA, Dvorkin M, Kulikov AS, Lesin VM, Nikolenko SI, Pham S, Prjibelski AD, Pyshkin AV, Sirotkin AV, Vyahhi N, Tesler G, Alekseyev MA, Pevzner PA. 2012. SPAdes: a new genome assembly algorithm and its applications to single-cell sequencing. J Comput Biol 19:455–477. doi:10.1089/cmb.2012.0021 PMC3342519 22506599

[15] Buchfink B, Reuter K, Drost HG. 2021. Sensitive protein alignments at tree-of-life scale using DIAMOND. Nat Methods 18:366–368. doi:10.1038/s41592-021-01101-x PMC8026399 33828273

[16] O’Leary NA, Wright MW, Brister JR, Ciufo S, Haddad D, McVeigh R, Rajput B, Robbertse B, Smith-White B, Ako-Adjei D, et al.. 2016. Reference sequence (RefSeq) database at NCBI: current status, taxonomic expansion, and functional annotation. Nucleic Acids Res 44:D733–D745. doi:10.1093/nar/gkv1189 PMC4702849 26553804

[17] Tisza MJ, Belford AK, Domínguez-Huerta G, Bolduc B, Buck CB. 2021. Cenote-taker 2 democratizes virus discovery and sequence annotation. Virus Evol 7:veaa100. doi:10.1093/ve/veaa100 PMC7816666 33505708

[18] Tisza MJ, Pastrana DV, Welch NL, Stewart B, Peretti A, Starrett GJ, Pang Y-YS, Krishnamurthy SR, Pesavento PA, McDermott DH, et al.. 2020. Discovery of several thousand highly diverse circular DNA viruses. eLife 9:e51971. doi:10.7554/eLife.51971 PMC7000223 32014111

[19] Tisza MJ, Varsani A, Petrosino JF, Cregeen SJJ. 2026. Cenote-taker 3 for fast and accurate virus discovery and annotation of the virome. Peer Community J 6:6. doi:10.24072/pcjournal.706

